# Beneficial Effects of Natural Bioactive Compounds from *Hibiscus sabdariffa* L. on Obesity

**DOI:** 10.3390/molecules24010210

**Published:** 2019-01-08

**Authors:** Oyindamola Vivian Ojulari, Seul Gi Lee, Ju-Ock Nam

**Affiliations:** 1 Department of Food Science and Biotechnology, Kyungpook National University, Daegu 41566, Korea; hoyeendahmolar@gmail.com (O.V.O.); lsg100479@naver.com (S.G.L.); 2 Institute of Agricultural Science & Technology, Kyungpook National University, Daegu 41566, Korea

**Keywords:** adipogenesis, bioactive compounds, fat accumulation, *Hibiscus sabdariffa*, lipase inhibition

## Abstract

Obesity is a condition associated with the accumulation of excess fat in the body, energy imbalance, lipogenesis, etc., which increases adipose tissue mass through adipogenesis and poses a health risk. Its prevalence has become an economic burden to the health care system and the world at large. One of the alternatives to tackling obesity involves the use of bioactive compounds. We critically examined the effects of *Hibiscus sabdariffa extract* (HSE) on various parameters associated with the development of obesity such as; the effect of HSE on body weight, the effect of HSE on lipid accumulation, cholesterol metabolism and plasma parameters, the inhibitory effect of HSE on pancreatic lipase, and the effect of HSE on adipocyte differentiation/adipogenesis. This review has gathered reports on the various anti-obesity effects of *H. sabdariffa* bioactive compounds in cell and animal models, as well as in humans. Available toxicology information on the consumption of *H. sabdariffa* revealed that its toxicity is dose-dependent and may cause an adverse effect when administered over a long period of time. Reports have shown that *H. sabdariffa* derived bioactive compounds are potent in the treatment of obesity with an evident reduction in body weight, inhibition of lipid accumulation and suppression of adipogenesis through the PPARγ pathway and other transcriptional factors.

## 1. Introduction

Obesity is a major public health problem associated with an increased incidence of metabolic diseases like type 2 diabetes, high blood pressure, heart disease, liver disease, kidney disease, gallbladder disease, and certain types of cancer [1]. The underlying cause of obesity can be attributed to the diet, genetic disorders, sedentary lifestyle, and psychological factors. The imbalance between energy intake and expenditure also causes the buildup of excess adipose tissue [2].

Several approaches have been made towards combating obesity, which include the clinical approach like surgical and lifestyle changes, exercising to induce weight loss and reduce fat accumulation, dietary approaches like caloric restriction, ketogenic diet and pharmacological approaches in which weight loss drugs have been developed. However, this approach has been found to be seasonal and not long-lasting [3], as it eventually creates a *yo-yo* effect. For a more lasting solution, research is leaning towards the use of bioactive compounds in plant materials as a novel therapy to combat obesity and its related diseases.

Recently, natural bioactive compounds such as flavonoids and phenols have been reported to be effective in the treatment of obesity [4,5,6,7]. Bioactive compounds in edible plants such as epigallocatechin gallate (EGCG) from green tea, nobiletin from citrus peel, curcumin from turmeric, resveratrol, pterostilbene from berries and anthocyanins from *H. sabdariffa* have been reported for their anti-obesity potential in both in-vivo and in-vitro studies [8,9,10,11,12]. *H. sabdariffa* also known as Roselle has a long history of usage as a beverage and folk medicine in places such as Thailand and, Nigeria, China, and India [13,14,15].

Studies on the phytochemical properties of *H. sabdariffa* showed that it has several health benefits and could be used as a potent material for the therapeutic treatment of various diseases [16,17]. Its chemical composition also showed that the therapeutic potency of *H. sabdariffa* could be traced to the presence of bioactive compounds. Bioactive compounds such as flavonoids (quercetin, luteolin, and its glycoside); chlorogenic acid, gossypetin, hibiscetin, phenols, some phenolic acids, anthocyanins such as delphinidin-3-sambubioside and cyanidin-3-sambubioside were detected as the main components in the aqueous extract of *H. sabdariffa* [18]. These bioactive compounds together or alone have been reported in many studies to possess potent antioxidant, anti-inflammatory, anti-carcinogenic effects and may also help control diabetes, prevent cardiovascular disease and obesity [19,20,21,22,23,24,25,26].

Reviews are available on the use of *H. sabdariffa*. However, reviews on *H. sabdariffa* and its bioactive compounds in relation to obesity are still insufficient. Previous reviews have focused on the phytochemical, pharmacological and toxicological properties of *H. sabdariffa*. [15,17,27,28]. Other comprehensive reviews documented by Wahabi et al., [29], Carjaval-Zarabal., [30], Hopkins et al., [31] Serban et al., [32] and Walton et al., [33] revealed the effect of *H. sabdariffa* in the treatment of hypertension hyperlipidemia and apoptosis. Its traditional use, nutritional composition, bioactive constituents, health benefits, and therapeutic use were examined by Riaz and Chopra [17], Singh et al., [34], and Ismail et al., [35]. A review by Herranz-Lopez et al., [36] focused on the multi-targeted molecular effect of hibiscus polyphenols on obesity management, Aziz et al., [37] has also reviewed its effect on serum lipids. Therefore, this present review takes into consideration available reports on the beneficial effects as well as toxicology of natural bioactive compounds in *H. sabdariffa* in relation to obesity therapy.

## 2. Natural Bioactive Compounds in *H. sabdariffa*

Bioactive compounds are compounds produced by plants that have pharmacological or toxicological effects in man and animals [38]. Various natural compounds have been identified to influence weight loss, fat accumulation, and avert diet-induced obesity. Hence, these products have been extensively consumed for the treatment of abdominal obesity and overweight [4,39,40].

**Figure 1:**
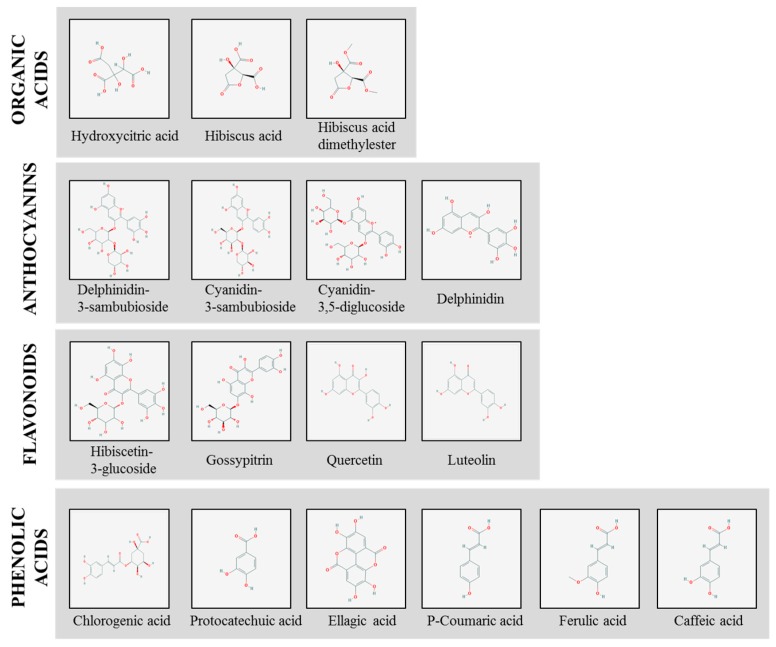
Chemical structural formula of major bioactive compounds in *H. sabdariffa*.

Many studies have shown the calyxes of *H. sabdariffa* to contain various compounds. The main bioactive compounds generally believed to be the active constituents responsible for the therapeutic effects of *H. sabdariffa* are organic acids, anthocyanins, flavonoids and phenolic acids, as seen in Table 1 and Figure 1 which shows their chemical structural formula.

Different studies (as shown in Table 2) have attributed the anti-obesogenic potential of *H. sabdariffa* extract (HSE) in obesity treatment to one or more specific compounds that it contains [11,57]. While some authors reported polyphenols [8,20,58] and anthocyanin [9,10] as the main active component in *H. sabdariffa*, another group of authors concluded in their report that other organic acids (hibiscus, dimethyl hibiscus and, hydroxycitric acids) contained in *H. sabdariffa* were responsible for the beneficial effects observed in their study [57]. The non-conformity of these reports could be due to the solvent used for extraction. Morales-Luna et al., [57] who compared both aqueous and methanolic extract of white and red varieties of *H. sabdariffa*, reported that aqueous extract of both varieties showed higher organic acid content compared to the methanolic extract, while polyphenols anthocyanin and flavonoids were higher in methanolic extract. However, some authors have suggested aqueous or aquueous-ethanolic solvent to be preferred for the extraction of anthocyanins, flavonoids and polyphenols from *H. sabdariffa* calyes [11,58,59,60]. Various reports on the effects of these compounds on obesity treatment will be highlighted under different sections of this review.

## 3. Effect of *H. sabdariffa* Extract (HSE) on Obesity

### 3.1. Effect of HSE on Body Weight

As aforementioned, obesity can be characterized by chronic energy imbalance and excessive body weight [2,69]. This disruption in energy homeostasis results in abnormal adipocyte differentiation, which is characterized by hyperplasia and hypertrophy of adipocytes [70]. The potentiality of polyphenols in *H. sabdariffa* to regulate energy metabolism and its beneficial effect on lipid management and weight loss as studied by several authors will be examined in this section.

Villalpando-Arteaga et al. [61] reported a reduction in body weight in relation to inhibition of fat accumulation of obese C57BL/6NHsd mice after treating with 33 mg/kg three times a week for 8 weeks. Impressively, Alarcon-Aguilar et al., [9] recorded a 9.6% decrease in body weight of obese Monosodium glutamate (MSG) mice with daily administration of 33.64 mg/kg/day for 8 weeks. A significant shift in body weight was noticed from the 7th week, which was attributed to a reduction in food with a consequent increase in liquid intake.

Herrara-Arellano et al., [25] who also reported a similar observation, attributed this result to the diuretic effect of *H. sabdariffa*. In spite of these observations, the author suggested that further long-term toxicity studies should be performed on this plant, especially since the previous study by Akindahunsi and Olaleye [71] have identified in their study that prolonged usage of this extract at a 15-dose level caused liver injury while the effect was mild at small dose levels (1–10). Carvajal-Zarrabal et al., [12] also reported that HSE administered at concentrations of 5% to 15% was effective in body weight reduction at intermediate and greater concentrations of 10% and 15% used in their experiment. In vitro and in vivo studies showed that HSE (or tea) inhibited the activity of a-amylase, blocking sugars and starch absorption, which may assist in weight loss [72,73]. Overall, in most studies, the ability of HSE to reduce body weight was attributed to *H. sabdariffa* polyphenols and flavonoids, through the inhibition of fat accumulation [9,61].

### 3.2. Effect of HSE on Lipid Accumulation, Cholesterol Metabolism and Plasma Parameters

Other factors are also involved in causing the onset of obesity. The major mark of obesity is abnormal or excessive fat accumulation in adipose tissue. The amount of excess fat in absolute terms, and its distribution in the body—either around the waist and trunk (abdominal, central or android obesity) or peripherally around the body (gynoid obesity) have important health implications [74]. The effect of *H. sabdariffa* on weight loss in early studies prompted further studies on its effect on lipid profile. Quite a number of studies have emphasized HSE as having an effect on inhibiting and/or reducing fat accumulation.

Bioactive compounds (polyphenolic and flavonoids) in *H. sabdariffa* have been reported to decrease oxysterols (a derivative cholesterol) in bile acid metabolism and block lipid accumulation in the liver [75]. In a study conducted by Carvajal et al., [12] on the modulation of fat absorption in rats by HSE, it was reported that HSE modulated fat absorption by increasing palmitic acid excretion in feces, accompanied by a decrease in triglycerides and cholesterol levels, including low-density lipoproteins (LDL) cholesterol. Another study carried out to evaluate the effects of HSE powder on the lipid profiles of individuals with and without metabolic syndrome (MS) showed that HSE significantly reduced glucose and total cholesterol levels, increased HDL levels, and triglycerides/HDL ratio in patients with MS [76]. In cholesterol-fed rabbits and high fructose-fed rats, HSE also decreased the number of oxidized LDL positive foam cells and total cholesterol and triglycerides concentrations in plasma [67]. According to Morales-Luna et al., [57], it was reported that 22.5 mg aqueous HSE of the white variety of Roselle prevented the increase in body weight of rats that were fed a high-fat fructose diet.

A more recent study investigated the effect of HSE in the reduction of fat tissue accumulation in high fat diet-induced obese C57BL/6NHsd mice [61]. A high-fat diet (HFD), along with reduced physical activity, induces excessive storage of triglycerides in adipocytes that leads to hypertrophy of the adipose tissue (AT). The study reported that HSE greatly diminished the accumulation of fat in the cytoplasm of hepatocytes. A significant reduction (*p* < 0.05) was observed in the gene expression of both transcription factors PPARγ and SREBP-1c in obese mice supplemented with HSE compared to obese mice. These authors claimed that HSE regulated the lipid homeostasis through SREBP-1c and PPARγ inhibition by blocking the increase of IL-1, TNF-α mRNA and lipoperoxidation and increased catalase mRNA; counteracting liver damage in an agonist-dependent manner. Hence, they concluded that HSE possesses an anti-steatogenic effect in the liver besides the anti-lipidemic and anti-obesogenic effects in the HFD-induced obese mouse model. Considering that fat accumulation is highly associated with obesity, accumulations of fat in organs have been a major concern in obesity management.

In liver steatosis, the anti-steatogenic effect of HSE on fatty liver (caused by fat accumulation in the liver) was recently conducted on humans. A clinical study conducted on patients with fatty liver within the age of 18–65 revealed that 2 HSE capsule-dose (1 g) after meals, 3 times a day significantly reduced the level of serum free fatty acid (FFA), exerting a beneficial effect on metabolic regulation, while improving the liver steatosis. However, this study observed no significant difference in the lipid profile except for FFA [66]. Furthermore, it was inferred in this study that polyphenol was mainly responsible for the clinical effect of HSE capsule and hence an increase in its dose could be more effective. A dose-dependent decrease in triglycerides levels, fatty acid concentrations and cholesterol contents of plasma lipids and liver lipids were observed in an in-vivo study of high-fat induced male Syrian hamsters fed with HSE [8]. HSE effectively inhibited lipid accumulation from fat-feeding and decrease the cholesterol in plasma and organs (liver) [8]. Affirmatively, these authors also attributed the effects observed in HSE-fed hamsters to the presence of polyphenols in HSE. Clinical studies on patients with metabolic syndrome, an obesity-associated disorder, further supported arguments of previous reports that polyphenols may be responsible for the therapeutic effect in HSE [20].

Based on these studies, it can be inferred that the presence of natural bioactive compounds such as polyphenols, flavonoids, and organic acids in HSE could be used as a preventive therapy in combating fat-induced obesity.

### 3.3. Inhibitory Effect of HSE on Pancreatic Lipase

Another strategy that has been proposed for the treatment of obesity is to inhibit pancreatic lipase, which consequently decreases lipid absorption in the intestine [77]. The underlying concept is that for any dietary fat being absorbed in the human intestine, the fat should be broken down enzymatically by the action of pancreatic lipase [78]. Pancreatic lipase activity is therefore widely considered as one of the most important indicators for the determination of the anti-obesity potential of natural products [79]. Orlistat, a potent, specific, and irreversible inhibitor of pancreatic and gastric lipases, is a weight-loss agent with a novel mechanism of action for the treatment of obesity. It inhibits gastric and pancreatic lipases in the lumen of the gastrointestinal tract to decrease systemic absorption (30%) of dietary fat [80]. However side effects such as diarrhea, fecal incontinence, flatulence, bloating and dyspepsia are commonly developed [11]. Due to these adverse effects, there has been a long-standing interest in discovering well-tolerated natural inhibitors for nutrient digestion and absorption.

The potential inhibitory activity against pancreatic lipase was also reported by examining the effect of HSE on fat absorption-excretion and body weight in rats [12]. Thus, continuous administration of *H. sabdariffa* polyphenols might improve obesity-related metabolic disorders in a similar manner to orlistat [36]. While this action may be viewed as a potential strategy in obesity management, its mechanism in obesity therapy is yet to be explored.

### 3.4. Effect of HSE on Adipocyte Differentiation (Adipogenesis)

Adipogenesis is the process by which cells differentiate into adipocytes. This process involves the conversion of preadipocytes into mature adipocytes with intracellular lipid accumulation [81]. Adipocytes are cells that primarily compose adipose tissue, specialized in storing energy as fat [82]. They play an important role in regulating adipokine secretion which promotes adipogenesis. Therefore, understanding the molecular mechanisms that regulate adipogenesis is important for exploring anti-obesity therapy [83].

Adipocyte differentiation is a critical phenomenon in the development and progression of obesity [84]. Adipocyte differentiation has been reported to be mainly mediated by the transcription factors PPARγ and C/EBPα [81]. These adipogenic transcription factors that are implicated to activate a number of genes induced during adipocyte differentiation is a master regulator of adipogenesis [85,86,87]. Hence, a down-regulation of PPARγ and C/EBPα has been viewed as a strategy to obstruct adipogenesis in adipocytes. Several studies have reported that extract of various medicinal plants attenuates expression of PPARγ and C/EBPα [10,61,88,89,90,91].

So far, only a few studies have reported and confirmed the effect of HSE on adipocyte differentiation. Kim et al., [68] first reported the effect of HSE treatment on adipocyte differentiation from 3T3-L1 preadipocytes and found that HSE blocked adipogenesis, possibly mediated through the suppression of adipogenic transcription factor expression. HSE treatment (100 mg/mL) inhibited the expression of major adipogenic transcription factors PPAR-γ and C/EBP-α, nuclear hormone receptors that regulate adipogenesis during differentiation. The authors further stated that this inhibitory effect of HSE on the transcription factors was target specific [68]. Further studies by Kim et al. [10], also confirmed that HSE can inhibit the adipogenic transcription factors by blocking the MAPK-mediated signaling pathway during adipocyte differentiation. They reported that HSE significantly decreased the mRNA levels of leptin (a hormone predominantly made by adipose cells that helps to regulate energy balance) during differentiation. Hence, suggesting that the effect of HSE on adipocyte differentiation was also mediated by the regulation of leptin [10].

A recent study performed on 3T3-L1 pre-adipocytes cells revealed that *H. sabdariffa* aqueous extract and *H. sabdariffa* polyphenols at concentrations 500 µg/mL and 10 µg/mL significantly inhibited adipogenic differentiation of pre-adipocytes [58]. These authors concluded that polyphenols contained in *H. sabdariffa* are mainly accountable for its effect on adipogenesis. Kao et al. [8], also showed that *H. sabdariffa* polyphenolic extract (HPE) was more efficient in suppressing adipogenesis than HSE as markers of adipocyte differentiation, while SREBP 1 was found to decrease in a concentration-dependent manner following treatment with both HSE and HPE. The authors reported in their study that after inducing maturation of preadipocyte, HPE suppressed the adipogenesis of mature adipocyte cells. This corroborates with the previously reported findings that polyphenols are the major active components in HSE that are responsible for its anti-obesogenic effect.

**Figure 2:**
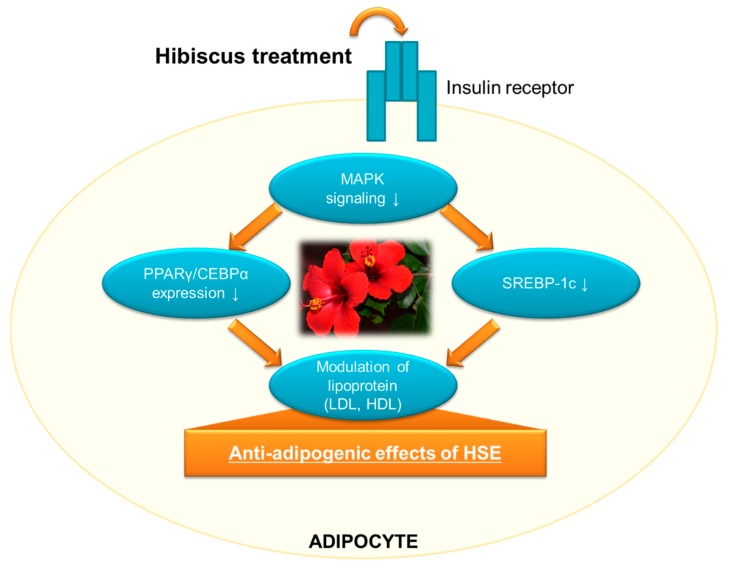
Anti-adipogenic effects on HSE through different pathways.

Based on the literature reviewed, *H. sabdariff’s* natural bioactive compounds such as polyphenols have shown good potential in modulating PPARγ and other transcriptional activities as shown in Figure 2, which makes it a potential therapy to inhibit adipogenesis and hence, combat obesity.

## 4. Toxicity of *H. sabdariffa*

Apart from the various beneficial effects of H sabdariffa, its safety for consumption as food or drug should also be addressed. Hibiscus sabdariffa have been reported to have a low degree of acute toxicity (Onyenekwe et al., [92]; Orisakwe et al., [93]; Ndu et al., [94]; Hopkins et al., [31]. Pharmacological studies identified that its co-administration with drugs may lead to increased side effects, toxicity or therapeutic failure (Johnson et al., 2013; Kolawole & Maduenyi, [95]; Ndu et al., [94].

Jabeur et al., [60] in their study used porcine liver primary cell culture (PLP2) to evaluate the hepatotoxicity of the HSE. They reported that no toxicity was observed in HSE up to the maximal tested concentration of 400 μg/mL. Non-cytotoxicity of Hibiscus sabdariffa aqueous extract has also been observed on normal fetal foreskin fibroblast (HFFF) cells (Khaghani et al., [96]. The doses of 250–1000 mg/kg/day HSE administered for 30days did not show any harmful effects organs system like liver, kidney, blood system, electrolytes, lipid, and carbohydrate metabolism of Wistar rats (Prommetta et al., 2006). However, high doses and prolonged usage have been reported to cause liver injury (Akindahunsi & Olaleye [71]; Fakeye et al., [97]). A high dose (300–2000 mg/kg) of HSE for 3 months was reported to have an adverse effect on liver enzymes, indicating that at very high doses, HSE could be hepatotoxic (Fakeye et al., [97]. Contrary to the previous statement, animal studies using male Wistar albino rats reported that high doses administration of HSE (4600 mg/kg) for 3 months did not cause toxic reactions, although negative effects on testes and sperm count were found (Orisakwe et al., [93]. Also, according to Onyenekwe et al., [92], no death was observed in mice fed with high doses of HSE (5000 mg/kg) for 14 days. Thus, these authors estimated the lethal dose to be greater than 5000 mg/kg.

## 5. Conclusion

Obesity is a common but often underrated condition that is globally considered to be a major public health concern that contributes significantly to the rate of morbidity and death. This review has shown the effectiveness of HSE on obesity-related parameters according to different experimental designs employed in various studies reviewed. Results have consistently reported that HSE consumption reduces body weight, lipid accumulation and total cholesterol metabolism in both animal and human studies. Its effectiveness in inhibiting pancreatic lipase and on adipocyte differentiation has also been frequently reported, thus confirming its therapeutic potential in obesity management.

*H. sabdariffa* extracts are generally considered to have a low degree of toxicity. However, some studies revealed that *H. sabdariffa* consumption at high doses may have some adverse effects. Hence more animal and clinical studies are required to determine safe doses of *H. sabdariffa* to balance the therapeutic and toxicological effects. Since clinical and animal studies have shown *H. sabdariffa* bioactive compounds to be effective in combating obesity, its regulated use as an active ingredient incorporated into diets at safe dosage is suggested.

The inconsistency of available studies on specific bioactive compounds responsible for anti-obesogenic properties of *H. sabdariffa*, as well as effective and safe doses, makes it necessary to carry out further studies for a more unanimous report.

## Figures and Tables

**Table 1:** Major bioactive compound in *H. sabdariffa*.

Bioactives	Chemical Formula	Studies on Obesity
**Organic acids**		
Hydroxycitric acid	C_6_H_8_O_8_	[41,42]
Hibiscus acid	C_6_H_6_O_7_	-
Dimethyl hibiscus acid	C_8_H_10_O_7_	-
**Anthocyanins**		
Delphinidin-3-sambubioside (hibiscin)	C_26_H_30_O_16_	-
Cyanidin-3-sambubioside (gossypicyanin)	C_26_H_30_O_15_	-
Cyanidin-3,5-diglucoside	C_26_H_30_O_15_	-
Delphinidin (anthocyanidin)	C_15_H_11_O_7_	[43]
**Flavonoids**		
Hibiscitrin (hibiscetin-3-glucoside)	C_21_H_20_O_14_	-
Gossypitrin	C_21_H_20_O_1__3_	-
Quercetin	C_15_H_10_O_7_	[44,45]
Luteolin	C_15_H_10_O_6_	[46]
**Phenolic acid**		
Chlorogenic acid	C_16_H_18_O_9_	[47,48]
Protocatechuic acid	C_7_H_6_O_4_	[49]
Ellagic acid	C_14_H_6_O_8_	[50,51]
P-Coumaric acid	C_9_H_8_O_3_	[52]
Ferulic acid	C_10_H_10_O_4_	[53,54]
Caffeic acid	C_9_H_8_O	[55,56]

**Table 2:** Studies on *H. sabdariffa* bioactive compounds and their anti-obesity effects.

Subject	Extraction Solvent	Effective Dose	Species	Observed Effect(s)	References
Mouse	Aqueous	33.64 mg/kg	Swiss Webste (CFW)	Suppressed body weight gain in Ob/MSG mice by 9.6% and reduces glycaemia	[9]
	Aqueous	33 mg/kg	C57BL/6NHsd	Reduced fat tissue accumulation, normalized the glycemic index as well as reduced dyslipidemia	[61]
Rat	Methanol	100 mg/kg, 200 mg/kg	Sprague-Dawley rats	Significantly reduced the plasma level of triacylglycerol cholesterol, and LDL/HDL risk ratio	[62]
	Methanol	200 mg/kg	Sprague-Dawley rats	HPE inhibited fat deposition, hyperglycemia, serum advanced glycation end-products (AGE)	[63]
	Aqueous and Methanol	3% of 750 mg (22.5mg)	Wistar Rats	Prevented increase in body weight and decreased adipocytes hyperplasia on rats fed a hypercaloric diet	[57]
	Ethanol	5%, 10%, 15% (*v*/*v*)	Sprague-Dawley rats	Prevented increase in body weight, triglyceride, LDL and total lipids were lowered	[12]
	Aqueous	100 mg/kg	Sprague-Dawley rats	Attenuated body weight gain, plasma leptin, cholesterol, triglycerol, LDL and systolic blood pressure	[64]
	Aqueous	500 mg/kg	Sprague-Dawley rats	Prevented the hypercholesterolemia induced by dietary fructose	[65]
Human	Aqueous	1 g HSE capsule-dose	In vivo (human trial)	Decreased serum FFA level of the HSE group and improved liver steatosis	[66]
	-	100mg (Capsule)	In vivo (clinical study)	Patients with metabolic syndrome showed a significant reduction in total cholesterol and glucose level, with increased HDL level	[67]
Cultured cells	Methanol	0.25 or 0.5 mg/mL	3T3-L1 pre-adipocytes cell	Suppressed differentiation and increased number of apoptotic cells in mature adipocytes	[8]
	Aqueous	2 mg/mL	3T3-L1 pre-adipocytes cell	Inhibition of lipid accumulation by reducing intracellular lipid droplet during adipogenesis	[10]
	Aqueous	100 mg/mL	3T3-L1 pre-adipocytes cell	Reduced the expression of major adipogenic transcription factors including PPARγ and C/EBPα that regulate adipogenesis	[68]
	Aqueous and ethanol	500 µg/mL aqueous extract and ethanol extract 10 µg/mL	3T3-L1 pre-adipocytes	Inhibited lipid accumulation and adipogenic differentiation of pre-adipocytes	[58]

## Abbreviations

Adipose tissue	AT
Epigallocatechin gallate	EGCG
Free fatty acid	FFA
High-density lipoproteins	HDL
*Hibiscus sabdariffa* extract	HSE
Interleukin-1	IL-1
Low-density lipoproteins	LDL
Messenger RNA	mRNA
Metabolic syndrome	MS
Not specified	NS
Peroxisome Proliferator-activated Receptor γ	PPARγ
Sterol regulatory element-binding protein	SREBP-1c
Tumor necrosis factor α	TNF-α
